# Patient safety culture awareness among healthcare providers in a tertiary hospital in Riyadh, Saudi Arabia

**DOI:** 10.3389/fpubh.2022.953393

**Published:** 2022-07-18

**Authors:** Abdulkarim Alsulami, Ashraf A'aqoulah, Nouf Almutairi

**Affiliations:** ^1^Department of Health Systems Management, College of Public Health and Health Informatics, King Saud bin Abdulaziz University for Health Sciences, Riyadh, Saudi Arabia; ^2^King Abdullah International Medical Research Centre, Riyadh, Saudi Arabia; ^3^Department of Health Informatics, College of Public Health and Health Informatics, King Saud bin Abdulaziz University for Health Sciences, Riyadh, Saudi Arabia

**Keywords:** patient safety, patient safety culture, patient safety awareness, healthcare providers, Saudi Arabia

## Abstract

Patient safety is a serious concern in the health care industry. To enhance patient safety, healthcare providers are expected to minimize accidental harm to patients and enhance the quality of patient-centered care. The main objective of this study is to explore the awareness of the patient safety culture among healthcare providers. It is further intended to assess key fields and factors that hinder patient safety adoption and determine the effects of demographic factors on healthcare providers' awareness of patient safety culture. This study applied a cross-sectional quantitative design. It was conducted in a tertiary hospital in Riyadh, Saudi Arabia. The participants consisted of all healthcare providers working in a specific tertiary hospital in Saudi Arabia. A random sampling technique was applied in this study. The study sample size was 409 participants. A valid and reliable questionnaire was used to collect the required data. The *T*-tests, ANOVA. And regression was used. The study found that there is a moderate level of patient safety culture awareness among healthcare providers. Moreover, the findings also revealed that the age group “31–40” showed statistically different awareness levels as compared to the “more than 50 years' age group” (*p* = 0.012). Also, this study has found that gender and education have a significant influence on the awareness level of patient safety culture while position and work area have no influence on the awareness level of patient safety culture among healthcare providers. Managers in healthcare institutions should develop speeder response plans and make them part of the patient safety culture. Institutions offering bachelor's degrees and postgraduate in nursing should pay more attention to the subject of patient safety. The government healthcare sector, together with the private healthcare sector, should continuously train healthcare providers on patient safety procedures to improve the patient safety culture. Healthcare providers should be encouraged to report errors made during diagnosis or treatments to avoid them in the future.

## Introduction

Patient safety is a serious concern in the health care industry globally. Harm prevention against patients and patient safety translates to a healthy patient safety culture and quality patient-centered care within many healthcare facilities. Aswat et al. observe that highly performing healthcare organizations have a healthy culture of patient care safety (1). Given this, it is important for healthcare organizations to create and maintain healthy patient safety cultures, not only to deliver quality patient-centered care but also for the betterment of the organizations' overall performance. Kumbi et al. define patient safety culture as “the values shared among organization members about what is important, their beliefs about how things operate in the organization, and the interaction of these within a work unit and organizational structures and systems, which together produce behavioral norms in the organization that promote safety” (2). Thus, to achieve a healthy patient safety culture, healthcare providers need to be aware of the expected organizational beliefs, values, and norms to achieve quality patient-centered care.

Globally, all healthcare providers, including nurses, are vulnerable to committing different medical errors, consequently causing patient safety to be a critical issue in the healthcare industry. For this reason, enhancing patient safety can significantly help to improve the general outcomes in every healthcare setting. Despite the probability of healthcare providers getting harmed being low globally (one out of 300) (3). Alnasser et al. observe that the costs due to medication errors are approximately 42 billion USD per year (4). In Saudi Arabia, specifically, a research study by Eldeeb et al. reveals that many healthcare practitioners, including nurses, have reduced levels of patient safety (5). Similarly, Khalil and his colleagues observe that the most serious events within the Saudi Arabia healthcare industry are related to allergic reactions due to medication errors (6). However, they are seldom formally reported. For this reason, the Saudi Arabia healthcare industry needs a complete overhaul regarding patient safety culture awareness among healthcare providers.

Different research studies have attributed the vulnerability of patients and healthcare providers within the Saudi Arabia healthcare industry to wrongful medication to different threats. Multiple studies show that the main reasons for wrongful medication and other threats occur due to the difficulty or inability of patients to communicate with physicians, language barriers, and the lack of comprehensive divulgence of both treatment plans and medical conditions (7). Also, a study by Parija and Adkoli established that the difficulty or inability of patients to communicate with physicians and language barriers are risk factors of harm in the administration of opioid medications within Saudi Arabia communities (8). Moreover, the patient-doctor interaction issue is also highlighted in a study by Alnasser et al. In Alnasser et al.'s study, it was established that most Saudi Arabia medical schools do not fully address the significance of patient-doctor communication in educational curricula (3). Furthermore, the healthcare industry in the region is highly dominated by foreign physicians.

There are many other breaches in the safety of patients in Saudi Arabia besides the difficulty or inability of patients to communicate with physicians and language barriers. For example, a study by Panagioti et al. attributes burnout among physicians to the occurrence of medical errors (9). Moreover, other research studies identify hygiene as an inhibiting factor to quality patient care and safety. For instance, research on gloving and handwashing practices by Basurrah and Madani established that poor handwashing practices among Saudi Arabia medical staff significantly compromise patient safety and the provision of quality patient care (10). These findings are also supported by Muller and his colleagues, who reveal that handwashing compliance practices among emergency department (ED) staff are approximately 29% (11). However, research by Alshammari et al. establishes that the compliance rate among the Saudi Arabia ED staff is at approximately 29% due to the departmental inability to implement hand hygiene protocols (12). Based on this finding, it is evident that Saudi Arabia's healthcare providers violate patients' safety protocols despite being cognizant of their implications.

Globally, patient safety is still a serious issue in the healthcare industry. To optimize patient safety, health care providers must minimize accidental harm to patients and enhance the quality of patient-centered care (13). Following the release of the Institute of Medicine's “To Err is Human” study in 1991, patient safety issues became more prominent. By that time, approximately 98,000 individuals perished each year due to hospital-related errors (1). The Institute of Medicine report emphasized the importance of patient safety and the need to hold clinicians and other health care providers accountable for irresponsible actions that put patients in jeopardy (14). Nevertheless, it was unclear how such healthcare inconsistencies would be addressed without causing conflict among healthcare workers, especially in terms of the reporting system (3). The Department of Health found that error prevention was a strategy that necessitated significant system-wide reforms centered on avoiding, identifying, and reducing harmful behavior in inpatients that could result in morbidity or mortality.

In population health, developing a healthy safety culture requires a thorough grasp of a society's cultural ideas, values, and norms. This knowledge helps healthcare executives identify attitudes and behaviors that should be encouraged, rewarded, or anticipated (3). For example, a critical examination of the situation in many healthcare systems has demonstrated the importance of focusing on organizational elements such as patient safety rather than individual wrongdoings. It was discovered that taking a cultural change approach to addressing patient safety issues yielded the best results in increasing healthcare quality and safety (4). Instead of blaming culprits, healthcare systems are urged to consider mistakes as opportunities for quality and safety advancements.

Culture is the sum of a population's attitudes, societal practices, experiences, and values that guide their overall behavior. The importance of being committed to discussing and learning from mistakes is emphasized in establishing safety culture (12–14). Such a culture recognizes the inevitability of mistakes and aggressively discovers hidden hazards while adding non-punitive ways to report errors and analyze potential negative outcomes. Cultural safety in the Saudi Arabia healthcare sector is similar to the concepts mentioned above. A research study by Eldeeb et al. reveals that many Saudi Arabia healthcare practitioners, including nurses, have reduced levels of patient safety. As a result, they do not officially file their reports on adverse events (11).

Similarly, research by Khalil and his colleagues observes that the majority of prevalent serious events within the Saudi Arabia healthcare industry are related to medication errors (4). However, they are seldom formally reported. These findings emphasize the need for improvements in the Saudi Arabia healthcare industry regarding patient safety culture awareness among healthcare practitioners.

More research works to assess the patient safety culture among Saudi Arabia health care practitioners, including pharmacists, nurses, specialists, physicians, and others. According to Alonazi et al., the nurses have a significant role in perpetuating a healthy Saudi Arabia patient safety culture (14, 15). Although patient safety is the business of eve healthcare are practitioners, nurses have a key role to play in the entire process of providing quality healthcare services.

Investigating nurses' perspectives on patient safety is a critical step in enhancing the safety of patients in Saudi Arabia. However, as already mentioned, research studies by Eldeeb et al. and Khalil et al. reveal that many Saudi Arabia healthcare practitioners, including nurses, have reduced levels of patient safety (4, 11). Their low formal reporting on adverse events and the serial revisions of existing patient safety policies (16). In addition, policy modifications and compliance to existing processes (17) are only regarded seriously when an organization requests international accreditation, according to research by Akologo and colleagues.

In Saudi Arabia, the frequency at which nurse practitioners incidents of medical errors in hospitals is low. Therefore, in Saudi Arabia, communication obstacles in reporting such incidents remain high. The major impediment is the high risk of disciplinary measures when nurses confess to committing a patient safety violation (1, 13, 14, 18). If the Saudi Arabia patient safety culture is to improve significantly, feedback communication is a critical aspect that must be improved. Furthermore, nurses stated that in circumstances where the patient safety concern did not substantially impact patients, the percentage of error reporting remained high (9). As a result of these findings, the tertiary healthcare system will be characterized by blame and anxiety, adversely harming the patient safety culture.

Research findings have repeatedly established the value of leadership in fostering a patient safety culture. In several studies (1, 17, 19, 20), pharmacists and nurses noted that support from leadership was critical in combating subsequent surgical and drug errors. Nurse and physician leaders, for instance, who admit the inevitability of mistakes show a positive attitude toward the problem as a system instead of seeing it as a mistake committed by an individual practitioner. Such leaders conduct detailed inquiries into the source of the error and take immediate action to address the core issues (19). Communication obstacles and fear of prosecution, for example, are cited as core reasons for significant patient safety issues by the management of numerous Saudi Arabia hospitals. As established by Alnasser et al., the Saudi Arabia healthcare industry is highly dominated by expatriates from non-Arabic speaking countries (3). As a result, there could be a communication problem between doctors and nurses, resulting in pharmaceutical errors being conveyed to hospitalized patients. Communication barriers may also prevent caregivers from fully comprehending medications, care plans, and follow-up treatment plans.

Various explanations have been highlighted in the literature as to why healthcare personnel has negative attitudes about patient safety culture. For example, the system has unfairly punished accused offenders of surgical and medication blunders (13, 15). The Ministry of Health has yet to adopt policies that examine the fundamental source of the problem and resolve it systematically. As a result, the rate of medical or surgical errors being reported is still quite low. Positive perceptions are connected with the frequency of errors and length of employment in places where medical and surgical mistakes are more commonly reported (1, 20, 21). Healthcare experts with extensive experience in the field report high rates of reporting incidences of errors. Moreover, healthcare institutions with a higher rate of patient safety issues appear to have more positive attitudes toward a culture of patient safety (1, 19, 21). Nevertheless, improving healthcare personnel's perceptions of patient safety requires more than just the rete of errors and experience.

Regrettably, medical research in Saudi Arabia lacks data on the extent of knowledge of issues affecting patient safety culture. Furthermore, it is uncertain what healthcare personnel expect regarding patient safety views (1, 22). Saudi Arabia currently has several policies in place to promote the establishment of a positive patient safety culture (1, 19). These policies, however, are seldom fully enforced, which explains why unfavorable impressions of patient safety persist. Based on this, the management of Saudi Arabia's healthcare facilities must demonstrate effective leadership to address all threats to the growth of patient safety. Specifically, they need to implement punitive measures against perpetrators of medical errors. This way, their attitude toward patient safety will improve, consequently impacting the safety culture in the Saudi Arabia health care industry.

## Research objectives

The main objective of this research is to assess the level of patient safety culture awareness among healthcare providers. Specific objectives of this study include identifying the main factors which hinder the implementation of patient safety, and determining demographic variables' effect on healthcare providers' awareness and perceptions of patient safety culture.

## Materials and methods

### Research strategy

The choice of the research strategy for this research is based on the research aim, objectives, and the existing research data and information on patient safety culture awareness among Saudi Arabia health care practitioners. Specifically, this research study applied a cross-sectional quantitative design. This is because it is the best research design method for measuring patient safety culture awareness among (healthcare providers. Additionally, since the researcher planned to select the participants using the inclusion and exclusion criteria, the cross-sectional quantitative design was the preferred research design strategy.

### Research area/setting

This research was conducted in a tertiary hospital in Riyadh, Saudi Arabia.

### Research participants

This research was based on the inclusion and exclusion criteria of the research participants. The inclusion criteria included healthcare workers in a tertiary hospital (Both clinical and non-clinical healthcare workers; participants' age was 20 years or above; the education level of participants a diploma or higher. The exclusion criteria included healthcare workers who do not work in a tertiary hospital; participants' age < 20 years; the education level of participants less than a diploma.

### Research sample & sampling technique

Based on the aims and objectives of this research, the randomization sampling technique was applied. The researcher selected this sampling technique to eliminate biases by giving every individual in the research sample an equal opportunity to be a participant. After subjecting the research sample to inclusion and criteria, 409 participants were identified.

### Data collection method, instruments used, measurements

The primary method of data collection for this research study was a valid and reliable questionnaire which was the Hospital Survey on Patient Safety Culture questionnaire. The questionnaire was anonymously distributed thru social networks to healthcare providers. All healthcare providers were briefed on the research aims and objectives beforehand.

The Hospital Survey on Patient Safety Culture questionnaire was adapted from the Agency for Healthcare Research and Quality (AHRQ) (22). The questionnaire elements were mildly modified to suit the researchers' intended respondents. The cultural sensitivity was also expertly evaluated.

The questionnaire was divided into two sections. The first section dealt with demographic information such as age, gender, years of experience, age, level of education, position in the hospital, and work area, while the other section included 12 aspects of patient safety culture. The 12 aspects covered in the second section include Patient Safety Rating, Communication Openness, Manager, Supervisor, Organizational Learning-Continued Improvement, Reporting Patient Safety Events, Number of Events Reported, Response to Error, Hospital Management Support for Patient Safety, Teamwork, Clinical Leader Support for Patient Safety, Hands-off and Information Exchange, Error Communication, and Staffing and Work Pace. The data for this study was collected *via* online platforms including social media networks.

### Statistical analysis

The patient safety culture and the cumulative score were obtained by calculating the findings of the domain after flipping the negative concerns based on the research aims and objectives of the research study. Higher scores indicated a more patient-centered culture. All variables on the scale had their standard deviations and means calculated, followed by *T*-tests and ANOVA. *T*-test was used to explore the association between level of awareness and gender variable because it has two categories. While one-way analysis of variance (ANOVA) was used to explore the association between level of awareness and demographic variables such as age group and education level because they have more than two categories

The aim was to discover the significance among the means. The logistic regression was applied in checking the influence of demographic variables on the level of awareness. The statistical significance level was set at 0.05.

## Results

### Descriptive statistics

The respondents of the study were 409 healthcare providers. Out of 409 respondents of the study, 6.6% were between the age of 20–30 years, 60.4% were between 31 and 40 years, 27.1% were between 41 and 50, and 5.9% were above the age of 50 years. As shown in Table 1, of the 409 participants, 143 (35%) were female, whereas 266 (65%) were male participants. Furthermore, Table 1 also contains statistics about the education status of the participants. The majority of the participants of the study (65%) had bachelor's degrees. Moreover, 21% had medical, 15.6% had nursing, whereas 36.4% had other clinical positions at the hospital.

**Table 1:** Frequency and percentage of participants' characteristics.

**Characteristic**	**Frequency**	**Percentage**
**Age**		
20–30 years	27	6.6
31–40 years	247	60.4
41–50 years	111	27.1
More than 50 years	24	5.9
**Gender**		
Female	143	35.0
Male	266	65.0
**Education**		
Diploma	35	8.6
Bachelor's degree	266	65.0
Postgraduate	108	26.4
**Position**		
Medical	86	21.0
Nursing	64	15.6
Other Clinical Position	149	36.4
Supervisor, Clinical leader,	52	12.7
**Senior leader, Manager, Executive**		
Support services	58	14.2
Total	409	100.0

### Components of patient safety awareness culture (PSAC)

Table 2 shows the mean scores and standard deviation scores of different components of patient safety awareness culture were calculated. To measure the healthcare providers' beliefs about the components, five points Likert scale was used (1, strongly disagree; 5, strongly agree) for the first seven components, whereas additional five points Likert scale (1, never; 5, always). Based on the mean score of 3.16, the value between 0.1 and 2.33 was considered a low level of awareness, the value between 2.34 and 3.66 was considered a moderate level of awareness, whereas the values above 3.66 were considered a high level of awareness. Staffing (*M* = 3.66) and communication about errors (*M* = 3.46) components had the highest mean scores. This means that healthcare providers' general staffing and their communication about errors were effective. On the other side response to error (*M* = 2.67) and hands-off and information exchange (*M* = 2.76) had the lowest mean scores. Overall, the mean score of patient safety awareness culture (PSAC) was 3.16. Thus, it can be concluded that there was a moderate level of patient safety awareness culture in the healthcare providers. Moreover, the mean scores of the other components of the scale, such as teamwork within units, organizational learning, management support for patient safety, communication openness, and reporting patient safety events, were reported as 3.04, 3.12, 3.20, 3.17, and 3.34, respectively.

**Table 2:** Mean and Std. Deviation of Domains of PSA.

**Domains**	**Mean**	**±SD**	**Level of Awareness**
Staffing	3.66	0.59	Moderate
Teamwork within units	3.04	0.45	Moderate
Organizational learning	3.12	0.53	Moderate
Response to errors	2.67	0.47	Moderate
Management support for patients' safety	3.20	0.55	Moderate
Hospital management support for patient safety	3.19	0.57	Moderate
Hands off and information exchange	2.76	0.56	Moderate
Communication about errors	3.46	0.66	Moderate
Communication openness	3.17	0.61	Moderate
Reporting patient safety events	3.34	0.80	Moderate
**All Domains**	3.16	0.23	Moderate

One of the major purposes of the study was also to check whether there is any influence of gender on the level of patient safety culture awareness. The analysis revealed that the mean score of the female and male groups were 3.20 and 3.14, respectively. The 143 female participants (*M* = 3.20, SD = 0.24) compared to the 266 male participants (*M* = 3.14, SD = 0.23) demonstrated significantly better safety awareness. Moreover, the group's mean scores are statistically significant because the *p*-value is 0.009.

Moreover, a one-way analysis of variance (ANOVA) was used to compare the variance in the group means within a sample. Age Group, level of education, position in the hospital, and work area variables were used as factors, whereas patient safety awareness culture (PSAC) was used as the dependent variable. The findings revealed in Table 3 that there is a statistically significant difference between the age groups as determined by one-way ANOVA (*p*-vale = 0.020). Similarly, a statistically significant difference was identified between the groups of the level of education *p*-value = 0.008). Whereas the results found no significant between means in position in the hospital and the same result was found in the work area domain.

**Table 3:** ANOVA table for the demographic data on the level of awareness.

**Variables**	**Source**	**Sum of squares**	**Degree of freedom**	**Mean Square**	**Sig.**
Age Group	Between Groups	0.526	3	0.175	0.020
	Within Groups	21.42	405	0.053	
Level of Education	Between Groups	0.521	2	0.261	0.008
	Within Groups	21.429	406	0.053	

As ANOVA test confirmed that the overall results were significant. Moreover, Tukey's Honest Significant Difference test was run to identify which group means are significantly different from another group's means. Tables 4, 5 show the multiple comparison analysis of the variable of age and education level. As the Tukey *post-hoc* test shows in Table 4, the 31–40 years age group showed statistically different awareness levels as compared to the “more than 50 years age group” (*p* = 0.012). The results of the Tukey *post-hoc* test in Table 5 show that the participants having diplomas showed statistically different awareness levels as compared to the participants having a bachelor's degree (*p* = 0.006).

**Table 4:** Multiple Comparison Analysis (*post-hoc*-Tukey HSD of Age Group).

**(I) Age**	**(J) Age**	**Mean Difference (I-J)**	**Std. Error**	**Sig.**	**95% Confidence**	
					**Lower Bound**	**Upper Bound**
31–40 years	20–30 years	0.04	0.04	0.798	−0.077	0.162
	41–50 years	0.02	0.02	0.785	−0.043	0.092
	More than 50 years	0.15^*^	0.04	0.012	0.024	0.278

* *The mean difference is significant at level ≤ 0.05*.

**Table 5:** Multiple Comparison Analysis (*post-hoc*-Tukey HSD of Education Level).

**(I) Education level**	**(J) Education level**	**Mean Difference (I-J)**	**Std. Error**	**Sig.**	**95% Confidence**	
					**Lower Bound**	**Upper Bound**
Bachelor's degree	Diploma	0.12^*^	0.041	0.006	0.029	0.224
	Postgraduate	0.03	0.026	0.455	−0.030	0.093

* *The mean difference is significant at level ≤ 0.05*.

## Discussion

This study is regarding patient safety culture awareness among health care practitioners based on current research and information. This study uncovered a few key findings on healthcare providers' perceptions and demonstrations the level of awareness of patient safety culture. This is because, while such findings are limited, they can provide significant data and information to the Saudi Arabia healthcare business and hospital administration about how to improve the level of patient safety culture among healthcare professionals for better patient care.

After calculating the mean scores and standard deviation scores of different components of patient awareness culture, it was discovered that there was a moderate level of patient safety awareness culture among healthcare providers. For all the components considered, all the means fell between 2.67 and 3.66, which was considered as a moderate level of awareness (2.34–3.66). Alshammari et al. did a research investigation that backs up the conclusions of this study. Alshammari et al. used a descriptive cross-sectional technique to assess the perspectives of healthcare practitioners about the culture of patient safety in their study (13). The study was carried out at four large Saudi Arabia hospitals. According to the findings of their research, the practice of patient safety is widely accepted among Saudi Arabia healthcare providers. They found positive relationships between study participants' profiles and patient safety dimensions. Furthermore, Aboshaiqah and colleagues discovered that nurses in Saudi Arabia had a positive opinion of patient safety culture in a survey targeted at finding out what they thought about it (23). According to the findings of this study, most healthcare professionals are aware of the necessity of patient safety culture, even if it has not yet been established in the system.

The findings also found that healthcare workers' general staffing and error communication is successful. Griffiths et al. found a link between nursing staffing and a number of patient safety outcomes in their investigation (24). Jahan and Siqiqqui also established that the doctor-patient relationship has a significant impact on health outcomes (25). For any relationship to thrive, communication is an essential component, particularly between a health practitioner and a patient. The author noted that in healthcare settings, communication is thorough and strong. Patient consistency and contentment improve with good communication. A physician's moral obligation is to answer all of the patient's questions and to make therapy and its effects as simple as possible. In line with the above studies, our study reveals that healthcare professionals in Saudi Arabia are aware of the importance of staffing and communication in promoting a culture of patient safety.

Although the research has established that healthcare practitioners in a tertiary hospital have a high mean of communication about errors (3.46), it contradicts the finding that they have a low mean of response to errors (2.67). This is because it is absurd for a healthcare practitioner to identify and communicate about a medical error and not respond to it on time. Nevertheless, this research finding regarding response to errors supports the finding by Alsafi et al., who earlier established a decrease in error reporting (49%) among Saudi Arabia healthcare providers. According to Alsafi et al., 49 percent of Saudi Arabia healthcare providers do not report formally medical errors if they (the errors) do not harm them (26). In terms of reporting patient safety events, the tertiary hospital has a high level of awareness (3.34). This is attributed to many factors, such as constant departmental training on the same and excellent patient counseling.

This research has found that hospital management supports patient safety culture (3.19). This, as already mentioned, could be due to constant staff training organized by hospital leaders. Globally, lack of hospital support has cost the healthcare industry approximately 42$ billion (3). The finding on the management of the tertiary hospital support for patient safety contradicts the findings by Alsulami and his colleagues, who established that approximately 44% of healthcare providers in Saudi Arabia tertiary healthcare setups do not understand the meaning of patient safety (27). This implies that the hospital's administrational support for patient safety is “a continuum of the healthcare system” (27). There are other reasons to explain why the management at the tertiary hospital has high support for patient safety. Research by Wittich et al. (31) attributes it to various factors, including a high therapeutic index (28). Additionally, Alsafi et al. observe that it could be attributed to the knowledge of various patient factors, including poor hepatic or renal function, polypharmacy, and impaired cognition (26). Alsafi et al. also acknowledge factors related to healthcare practitioners.

Demographic variables such as gender, age, education, hospital position, and work location had a low to moderate impact on the level of patient safety culture awareness among healthcare personnel, according to the findings of this study. There was a statistically significant difference between the age groups, according to the findings. It, therefore, means that age is a component of this study, affecting how healthcare practitioners perceive patient safety culture in Saudi Arabia. A research study by Jabarkhil et al. attributes this to their ability to handle strenuous workloads, workplace stress, and depression more easily (27). We can conclude that for the age category “31–40 years,” for every addition of a year, the awareness level increases. Similarly, for the age category “41–50 years,” the awareness level increases significantly. However, the category “More than 50 years” is taken as a reference category, and all other age categories are compared with it. According to Jabarkhil, this age categorization is prone to old age diseases such as mental loss (27). Coupled with workplace workloads, stress, and depression, they may not be able to recognize the violation of the expected patient safety protocols.

Another demographic component examined in the study is the influence of gender on the level of patient safety culture awareness. The findings show that the level of patient safety culture awareness among healthcare providers is strongly influenced by the age of the healthcare providers. Since the *P*-value was <0.05 (level of significance), the group's mean scores were statistically significant. Females demonstrated a significantly better safety awareness as compared to males. This could be attributed to the caring nature of females, which makes them more sensitive to patient safety than their male counterparts. Gender significantly directly affects both managerial and professional functions (29). The research findings further show that for different employee positions within the tertiary hospital, various PSAC aspects do not significantly relate to their General Patient Safety Perception. The position does not influence the “patient safety culture” awareness among healthcare providers.

In terms of the level of education, the participants having diplomas showed statistically different awareness levels as compared to the participants having a bachelor's degree (*p* = 0.006). It indicates that diploma courses in Saudi Arabia give more attention to patient safety practices as compared to bachelor's degrees and postgraduate. Negligence could originate from an assumption that since the students are at a higher level of learning, they need to be taught more advanced concepts.

According to the 2021 report by the World Health Organization, the organization is committed to providing useful information on medication safety, polypharmacy, high-risk situations, and transitional care (29). However, relevant research applicable for Saudi Arabia healthcare providers is lacking. In research conducted by Alenezi and his colleagues aiming to evaluate the perception of healthcare providers in accordance with their facility's “patient safety culture,” it was established that hospitals need to strengthen to enhance patient safety culture (30). For improving patient safety culture, Hospital managers need to focus on improving factors such as conducting training programs to reduce human-related incidents; enhancing safety monitoring and measuring system; Learning from the successful experience of other hospitals; Digitalization of healthcare management; Keeping patients informed to monitor their own treatment; improving communication among healthcare providers; focusing on hand hygiene procedure.

## Limitation

This research was conducted in one tertiary healthcare organization in Saudi Arabia. For this reason, the findings here are not entirely applicable to the whole Saudi Arabia healthcare industry. In addition, distributing the data collection tool was done *via* social networks or online platforms which means the healthcare providers who are not active in social networks did not get enough chance to participate in this study.

## Conclusion

The findings of this study revealed that healthcare providers have a modest level of “patient safety culture” awareness. Moreover, the findings also revealed that gender and education have a significant influence on the awareness level of “patient safety culture” among health care practitioners. On the other hand, position and work area have no influence on the level of “patient safety culture” awareness among healthcare providers. The results, therefore, affirm the first hypothesis that there is a moderate level of “patient safety culture” awareness among healthcare providers.

Moreover, the age of healthcare providers in the tertiary hospital has a statistically significant influence on the level of “patient safety” culture awareness. Specifically, this research has established that healthcare providers within the age of 31–40 are aware of the importance of patient safety culture (p = 0.001). This may be attributed to their ability to handle strenuous workloads, workplace stress, and depression more easily. Thus, this study has established that the age of the healthcare providers significantly influences the level of “patient safety culture” awareness among healthcare providers.

Based on the results of this study, there are several recommendations for action have been developed. Healthcare institutions should develop speeder response plans and make them part of the patient safety culture. Institutions offering Bachelor's degrees and postgraduate in nursing should pay more attention to the subject of patient safety. The Healthcare wing of the government, together with healthcare institutions, should continuously train healthcare professionals on the importance of “patient safety culture” and the various ways of responding to alarms. Health professionals should be encouraged to report errors made during diagnosis or treatments to improve patient safety.

## Data availability statement

The raw data supporting the conclusions of this article will be made available by the authors, without undue reservation.

## Ethics statement

The studies involving human participants were reviewed and approved by the Institutional Review Board (IRB) of King Abdullah International Medical Research Centre, Riyadh, Saudi Arabia. The patients/participants provided their written informed consent to participate in this study.

## Author contributions

AA and AA'a: conceptualization and methodology. AA, NA, and AA'a: formal analysis and data curation. AA: writing—original draft preparation. AA'a: reviewing and editing and project administration. All authors contributed to the article and approved the submitted version.

## Funding

The publication fees covered by King Abdullah International Medical Research Centre, Riyadh, Saudi Arabia.

## Conflict of interest

The authors declare that the research was conducted in the absence of any commercial or financial relationships that could be construed as a potential conflict of interest.

## Publisher's note

All claims expressed in this article are solely those of the authors and do not necessarily represent those of their affiliated organizations, or those of the publisher, the editors and the reviewers. Any product that may be evaluated in this article, or claim that may be made by its manufacturer, is not guaranteed or endorsed by the publisher.
